# Rivers shape population genetic structure in *Mauritia flexuosa* (Arecaceae)

**DOI:** 10.1002/ece3.4142

**Published:** 2018-06-11

**Authors:** Nilo L. Sander, Francisco Pérez‐Zavala, Carolina J. Da Silva, Joari C. Arruda, Maria T. Pulido, Marco A. A. Barelli, Ana B. Rossi, Alexandre P. Viana, Marcela S. B. Boechat, Christine D. Bacon, Angélica Cibrián‐Jaramillo

**Affiliations:** ^1^ Laboratório de Ecologia da Paisagem e Etnobiologia Centro de Pesquisas em Limnologia, Biodiversidade e Etnobiologia do Pantanal Universidade do Estado de Mato Grosso Cáceres Mato Grosso Brazil; ^2^ Programa de Pós‐Graduação em Biodiversidade e Biotecnologia da Amazônia legal Rede Bionorte Cuiabá Mato Grosso Brazil; ^3^ Laboratorio Nacional de Genómica para la Biodiversidad (Langebio) Unidad de Genómica Avanzada Centro de Investigación y de Estudios Avanzados del Instituto Politécnico Nacional Irapuato Guanajuato Mexico; ^4^ Laboratorio de Etnobiología Universidad Autónoma del Estado de Hidalgo Hidalgo México; ^5^ Laboratório de Melhoramento Vegetal Universidade Estadual do Norte Fluminense Campos dos Goytacazes Rio de Janeiro Brazil; ^6^ Department of Biological and Environmental Sciences University of Gothenburg Göteborg Sweden; ^7^ Gothenburg Global Biodiversity Center Göteborg Sweden

**Keywords:** Amazonia, anthropogenic effect, Arecaceae, gene flow, palm, rivers

## Abstract

The *Mauritia flexuosa* L.f. palm is known as the “tree of life” given its importance as fundamental food and construction resources for humans. The species is broadly distributed in wet habitats of Amazonia and dry habitats of the Amazon and Orinoco river basins and in the Cerrado savanna. We collected 179 individuals from eight different localities throughout these habitats and used microsatellites to characterize their population structure and patterns of gene flow. Overall, we found high genetic variation, except in one savanna locality. Gene flow between populations is largely congruent with river basins and the direction of water flow within and among them, suggesting their importance for seed dispersal. Further, rivers have had a higher frequency of human settlements than forested sites, contributing to population diversity and structure through increased human use and consumption of *M. flexuosa* along rivers. Gene flow patterns revealed that migrants are sourced primarily from within the same river basin, such as those from Madeira and Tapajós basins. Our work suggests that rivers and their inhabitants are a critical element of the landscape in Amazonia and have impacted the dispersal and subsequent distribution of tropical palm species, as shown by the patterns of genetic variation in *M. flexuosa*.

During my residence in the Amazon district I took every opportunity of determining the limits of species, and I soon found that the Amazon, the Rio Negro and the Madeira formed the limits beyond which certain species never passed. The native hunters are perfectly acquainted with this fact, and always cross over the river when they want to procure particular animals, which are found even on the river's bank on one side, but never by any chance on the other. On approaching the sources of the rivers they cease to be a boundary, and most of the species are found on both sides of them.

(Alfred .R. Wallace, 1852)

## 1 INTRODUCTION

Environmental and geographic features of the landscape are crucial in shaping the population genetic structure and demography of plants. The impact of rivers on population structure in the Amazonia has been documented in birds, small mammals, invertebrates (e.g., Aleixo, 2006; Colwell, 2000; Pellegrino et al., 2005; Ribas, Aleixo, Nogueira, Miyaki, & Cracraft, 2012; Vallinoto et al., 2006), and in trees and understory plant species (Huaman & Matthies, 2008; Nazareno, Dick, & Lohmann, 2017; Schleuning et al., 2011; Stevenson, 2007; Zhang, Zheng, & Song, 2007). Nonetheless, the extent to which rivers impact fine‐scale population genetic patterns and in particular, how factors such as the direction of river flow structure populations, are unknown.

*Mauritia flexuosa* is a conspicuous, widespread plant distributed most of South America. This species is long‐lived and dioecious, and its stems can reach up to 40 m in height (Delgado, Coutourier, & Mejia, 2007). The species is likely pollinated by beetles (Barfod, Hagen, & Borchsenius, 2011) and wind (Rosa & Koptur, 2013). Seeds are dispersed by a variety of mammals (Acevedo‐Quintero & Zamora‐Abrego, 2016) and fruits are capable of floating (Moegenburg, 2002). *Mauritia flexuosa* generally occurs below 1,000 m over sea level throughout Bolivia, Brazil, Colombia, Ecuador, French Guiana, Guyana, Peru, Surinam, and Venezuela. Its ample distribution comprises populations of thousands of individuals forming oligarchic forests (Peters, Balick, Kahn, & Anderson, 1989) and palm swamps, known as Aguajes (Peru), Buritizais (Brazil), or Morichales (Colombia, Venezuela), and are found in both rainforest and savanna biomes.

It is increasingly evident that pre‐Columbian and modern peoples that live along rivers have impacted the genetic patterns of forests within and among basins (Piperno et al., 2015; Stahl, 2015). Although there is no evidence of domestication of *M. flexuosa*, it is widely used by indigenous groups and local communities along rivers, who refer to it as the “tree of life” because it provides a variety of resources and it is consumed daily as a food staple (Barros & Da Silva, 2013). This palm is also used for raw material for construction and for different handicrafts (Santos & Coelho‐Ferreira, 2011) and its fruits, leaves, and seeds are sold widely in markets (Gilmore, Endress, & Horn, 2013). *Mauritia flexuosa* has been termed a “hyperdominant” species (Steege et al., 2013), in which population densities are five times higher than expected by chance, and that has recently been attributed to human use and tending practices associated to its use (Levis et al., 2017; Rull & Montoya, 2014).

Previous genetic studies of *M. flexuosa* using ISSR markers show high genetic diversity compared to other plants with similar life history traits (Gomes et al., 2011; Rossi et al., 2014). Chloroplast markers used to characterize *M. flexuosa* in different river basins revealed low nucleotide diversity within populations from the Brazilian savannas, which was interpreted as range retraction followed by population subdivision during the cold and dry periods of the Quaternary glacial periods (de Lima, Lima‐Ribeiro, Tinoco, Terribile, & Collevatti, 2014). These studies begin to provide information on the genetic structure of *M. flexuosa*, yet the genetic variation and population structure of *M. flexuosa* across different river basins remain to be tested more explicitly.

Our main research questions are whether rivers in Amazonian forests are facilitators or barriers to gene flow, whether population genetic structure is maintained in populations throughout river basins, and if recruitment is associated with river flow. We address these questions using microsatellite markers across different river basins in tropical forests and savanna sites, and we also discuss the impact of human river inhabitants in the generation of recent population structure of this palm species.

## 2 MATERIALS AND METHODS

### 2.1 Collection sites and sampling

**Figure 1:**
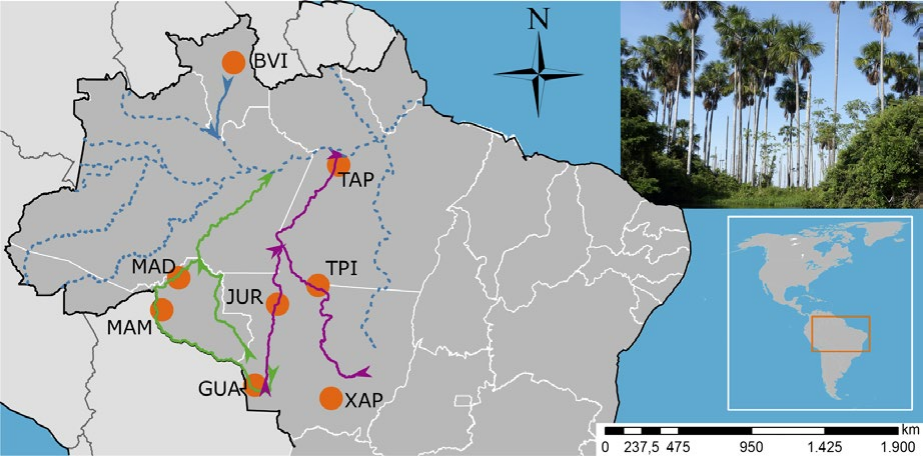
Map of the locations sampled in this study: the orange circles are the sites of the sampled populations. In purple, the Tapajós basin that includes the Juruena (JUR), Tapajós (TAP), and Teles Pires (TPI) rivers; in green the Madeira basin which include the Guaporé (GUA), Madeira (MAD) and Mamore (MAM) rivers; in solid blue we mark the closest river to the Boa Vista (BVI) population. In dashed lines are other rivers of the Amazonia. The arrows show the direction of the water flow. Chapada dos Guimarães (XAP) population is approximately 25 km to any other river

Plants were sampled from two of the major river basins in Amazonia—the Madeira and the Tapajós (Figure 1). The Madeira basin includes the Guaporé (GUA), Madeira (MAD), and Mamore (MAM) rivers; and the Tapajós basin includes the Juruena (JUR), Tapajós (TAP), and Teles Pires (TPI) rivers (Figure 1). Additional collections were also made from the Brazilian savanna (hereafter Cerrado) sites: Boa Vista (BVI; Roraima State) and Chapada dos Guimarães (XAP; Mato Grosso State), although these are limited in their representation of this wide geographic distribution. Sampling different environments (moist tropical forest, Cerrado) allows for contrasting levels of population diversity and structure. Given the broad distribution of this species (Brazil, Bolivia, Colombia, Ecuador, French Guiana, Guiana, Peru, Surinam, and Venezuela) we collected from sites that were at least 300 km away from each other in places that we considered representative of the region (Figure 1). Leaves were collected from an average of 22 reproductive individuals per site and sampled at least 100 m within the same location to avoid spatial autocorrelation and to increase the amount of information per population in microsatellite amplification, for a total of 179 samples distributed across eight sites in Amazonia (Figure 1; Table 1).

**Table 1:** Levels of genetic variation per sampling locality

Locality (population code)	*N*	*N*_a_	*H*_e_	*H*_o_	*F*
		Mean (*SE*)			
Rio Teles Pires, Alta Floresta, Mato Grosso (TPI)	24	6.5 (0.52)	0.70 (0.03)	0.63 (0.07)	0.08 (0.10)
Rio Juruena, Juruena, Mato Grosso (JUR)	22	5.7 (0.33)	0.70 (0.04)	0.59 (0.07)	0.13 (0.10)
Rio Tapajós, Santarém, Pará (TAP)	23	6.7 (0.58)	0.70 (0.03)	0.60 (0.06)	0.13 (0.09)
Rio Guaporé, Vila Bela da Santissima Trindade, Mato Grosso (GUA)	22	5.8 (0.39)	0.73 (0.02)	0.56 (0.09)	0.24 (0.12)
Rio Mamoré, Guajará‐Mirin, Rondônia (MAM)	22	7 (0.73)	0.74 (0.02)	0.62 (0.07)	0.17 (0.08)
Rio Madeira, Porto Velho, Rondônia (MAD)	21	5.9 (0.43)	0.69 (0.02)	0.59 (0.07)	0.15 (0.10)
Boa Vista, Roraima (BVI)	24	6.2 (0.47)	0.72 (0.02)	0.55 (0.08)	0.22 (0.12)
Chapada dos Guimarães, Mato Grosso (XAP)	21	4.6 (0.54)	0.63 (0.05)	0.60 (0.09)	0.05 (0.13)

*N* = number of samples evaluated; *N*_a_ = number of alleles; *H*_e_ = expected heterozygosity; *H*_o_ = observed heterozygosity, *F* = fixation index.

### 2.2 Microsatellite amplification

Leaves were collected in the field and stored in silica gel. DNA was extracted following the manufacturer's protocol of the Wizard Genomic DNA Purification kit (Promega, Madison, WI, USA). We selected 10 microsatellites previously designed for *M. flexuosa* (Federman, Hyseni, Clement, & Caccone, 2012; Menezes et al., 2012; Table S1) based on consistency of amplification. PCR conditions for all primers in individual reactions were 94°C for 5 min; 35 cycles of 94°C for 1 min, 62°C for 1 min, and 72°C for 1 min; then 72°C for 2 min. Amplification products were genotyped using capillary electrophoresis system (7.5 kW for 120 min; Advanced Analytical, Ankeny, IA, USA), together with standardized markers containing fragments of 35 and 500 bp and 75–400 bp DNA ladder in a single well to determine the size of the amplified fragments.

### 2.3 Genetic diversity and population genetic structure

MICRO‐CHECKER v 2.2.3 was used to correct genotypes for null alleles, scoring errors, and allelic dropout (van Oosterhout, Hutchinson, Wills, & Shipley, 2003). LOSITAN was used to test for neutrality in each locus with 1,000,000 simulations and a 99.5% confidence interval using both stepwise and infinite mutation models (Antao, Lopes, Lopes, Beja‐Pereira, & Luikart, 2008). To test for biases in the sample sizes and large distribution of this species, we estimated allelic richness by rarefaction for all populations using the *Vegan* v 2.4‐6 package (Oksanen, Kindt, Legendre, O'Hara, & Stevens, 2011) in the R statistical platform (R Core Team, 2014). Genetic diversity was calculated by assessing the number of alleles per locus, observed heterozygosity (*H*_o_), expected heterozygosity (*H*_e_), and the inbreeding coefficient (*F*) using Arlequin v 3.5 (Excoffier, Laval, & Schneider, 2005). We measured pairwise population genetic structure with *F*_ST_ (Wright, 1949) and *R*_ST_ (Slatkin, 1995), also using Arlequin. *R*_ST_ was used to complement *F*_ST_ as it is less sensitive to the fast mutation rate reported in microsatellites (Holsinger & Weir, 2009). We visualized pairwise *F*_ST_ and *R*_ST_ in a heat map using the R package *lattice* v 0.20 (Sarkar, 2015). Finally, given the large geographic scale of our samples and potentially confounding signals from isolation by distance (IBD; Meirmans, 2012), we estimated IBD between all sampling sites and within Basins, using the *adegenet* v 2.0.0. R package (Jombart, 2008).

To calculate regional and within‐population genetic diversity from different river basins and different regions, a Molecular Analysis of Variance (AMOVA) was conducted using the sum of squares size difference (*R*_ST_). The eight collection sites (GUA, MAD, MAM, JUR, TAP, TPI, BVI, and XAP) were divided into four groups based on major geographic areas: Madeira basin (GUA, MAD, and MAM), Tapajos basin (JUR, TAP, and TPI) and BVI and XAP. Significance was tested using 1,000 permutations with a 95% confidence interval. Population genetic structure was also measured using the Bayesian assignment method STRUCTURE v 2.3.4, which uses genotypes to assign individuals to a genetic group without a priori assumptions of populations (Pritchard, Stephens, & Donnelly, 2000). We used the admixture model and a correlated model with a burn‐in length of 100,000 steps with 2,000,000 replicates. We tested the number of distinct genetic clusters (populations; *K*) present in the data set from 1 to 10 using 20 iterations per *K*. We used a maximum of ten populations to allow for the possibility that a sampled location is substructured into more than one population. We used the Δ*K* method of Evanno, Regnaut, and Goudet (2005), implemented in STRUCTURE Harvester v 0.6.94, to determine the most likely number of genetic clusters *K* given our data (Earl & Von Holdt, 2011).

We also employed a graph theoretical framework to estimate population genetic summary statistics and to visualize the network of gene flow among populations that presumably results from both historical and contemporary history (Dyer & Nason, 2004). We defined each original sampling locality as a node and an alpha of .01 as the significance level to test edge retention, in the R package *popgraph v* 1.4 (Dyer & Nason, 2004). Additionally, to evaluate the direction of river water flow and its impact on gene flow patterns, we calculated migration rates among all sampled localities using BayesAss+ (Wilson & Rannala, 2003), which is a method that estimates immigration rates of a population with respect to all other populations, based on the analysis of genotypes using coalescent theory. Values closer to one indicate that individuals in that population are a result of self‐recruitment, while values closer to zero suggest that a population comprises migrants from other populations.

Finally, given the possibility of one or several founder events as a result of long‐distance seed dispersal by river water currents or by human use, we tested for reduction in population size using Wilcoxon sign‐rank test implemented in Bottleneck v 1.2.02 (Cornuet & Luikart, 1996). Under a model of mutation‐drift equilibrium, populations that have experienced a recent reduction in effective population sizes may present higher observed than expected heterozygosity (Maruyama & Fuerst, 1985). Although various models exist for microsatellites (Putman & Carbone, 2014), the SMM mutation model can implement equal probability of gaining or losing repeats, therefore accounting for homoplasy. We used the SMM model at 100%; the two‐phase mutation model allows for mutations of a larger magnitude than SMM but retains the mutation model and was used at 70% (Di Rienzo et al., 1994).

## 3 RESULTS

### 3.1 High genetic variation in *Mauritia flexuosa*

**Figure 2:**
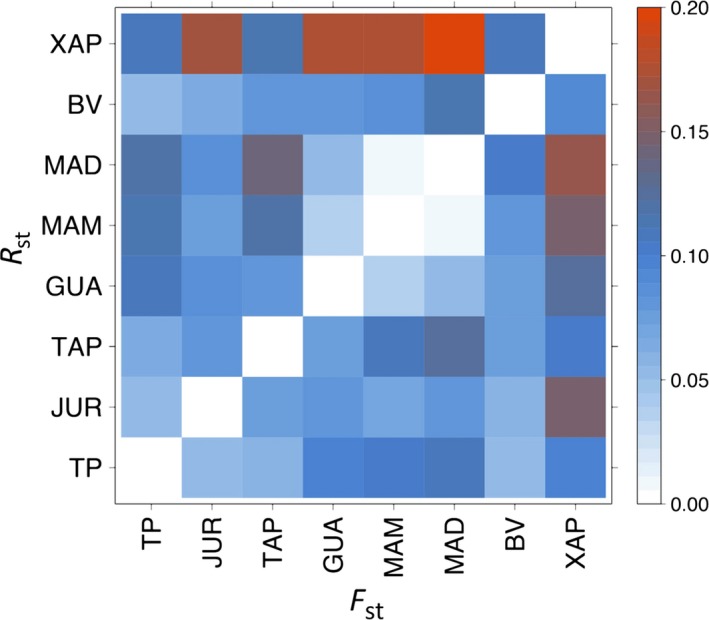
Population genetic structure of *Mauritia flexuosa* as measured by *F*_ST_ and *R*_ST_

**Figure 3:**
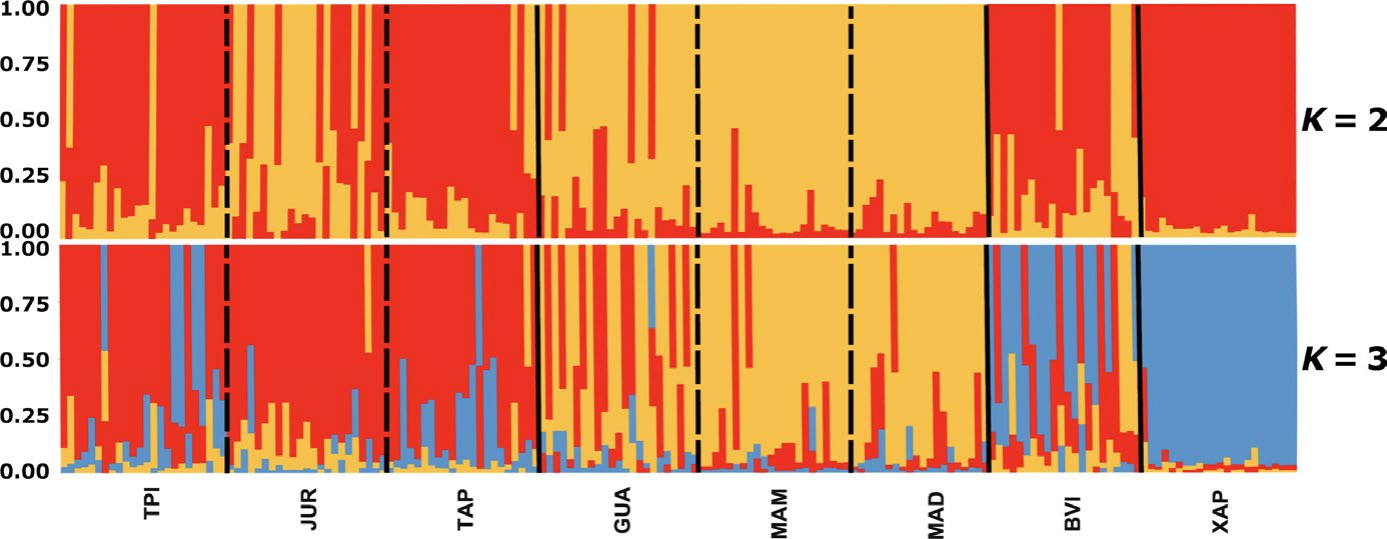
Inferred population structure for *K *=* *2 and *K *=* *3. Continuous lines represent divisions between basins and dashed lines divisions within the basin

No genotyping errors or null alleles were inferred using MICRO‐CHECKER. Eight pairs of loci were in linkage disequilibrium (Table S2), all populations deviated from Hardy–Weinberg Equilibrium with the exception of XAP (Table S2). Rarefaction estimates of allele richness in all populations showed that 70% of all possible alleles were sampled for all populations except for XAP (Table S2). A total of 67 alleles were identified from the 10 loci sampled, ranging from six (MF28) to 16 alleles per locus (MF14 locus). The mean of number of alleles for each population ranged from to 4.6 (XAP) to 7.0 (MAM; Table 1, Tables S2 and S3). Only one locus (MF13) showed signatures of positive selection using both mutation models (stepwise and infinite allele models) with a *p* = .999, although a comparison of genetic structure results with and without it were similar, and the locus was maintained in the analysis. Expected heterozygosity ranged from 0.63 in XAP to 0.74 in MAM. The observed heterozygosity ranged from 0.55 (BVI) to 0.63 (TPI; Table 1, Table S2). The fixation index ranged from 0.05 in XAP to 0.24 in BVI (Table 1). Overall, most sampled localities shared alleles, shown by the number and distribution of allele frequencies (Table S3), where the MAM population had the highest number of private alleles, and GUA and XAP had no private alleles (Table S3). We found IBD with marginally significant values of *r* = .42 (*p* = .02) among all populations, but none within basins (Table S2). Low values of pairwise *F*_ST_ (mean 0.08) and *R*_ST_ (mean 0.09) suggested low population structure, and even lower within river basins (Figure 2; Table S4), showing more connectivity within them than between them. The XAP site from the Cerrado was inferred as the most distinct and genetically differentiated from all other sites (Figure 2). Congruent with *F*_ST_, our AMOVA results showed that 78% of the variation is found within individuals and 4.3% of the variation among populations (Table S5). Also congruent with high levels of gene flow among populations, the most likely number of populations inferred from our data was three (Δ*K* = 3; Figure 3). In *K *=* *3, the Madeira basin (GUA, MAM, and MAD) and the Tapajós basin (TPI, JUR, and TAP) individuals from Amazonia are assigned into two clusters. The XAP Cerrado population was inferred as an independent genetic cluster, together confirming the results obtained by *F*_ST_ and *R*_ST_. BVI individuals had either admixed genotypes or shared ancestry within the Tapajós basin cluster. The population graph analysis showed that populations from the same river basin are highly connected, as in the case of JUR and TPI rivers that flow into the TAP (Figure S1) and the GUA, MAD, and MAM rivers that are part of the same basin. Our results showed some genetic connectivity between XAP and BVI, and it is clear that its genetic diversity is lower than the rest of the populations sampled (Figure S1), although its smaller sample size may affect this result (rarefaction; Table S2). We did not recover evidence of genetic bottlenecks in any site except for TAP (SMM 0.01).

We explored the exchange of migrants in finer detail to test if direction of river flow was a factor in *M. flexuosa*'s genetic structure. BayesAss+ results (Figure S1, Table 2) showed that populations with the lowest proportion of self‐recruitment were GUA (0.79) and MAM (0.67) and thus more open to outside immigrants. The MAD population received the largest proportion of migrants from MAM in the direction of river flow (0.24). However, most populations show high levels of self‐recruitment, with XAP and JUR being the highest (both 0.91) and thus directionality of rivers was harder to test.

**Table 2:** Bayesian assessment of migration within and among sampling localities implemented in BayesAss+. For each sampling locality, numbers are the mean proportion of individuals for each source locality. Boldface terms along the diagonal are proportion of non‐migrants (self‐recruitment). Above and below the diagonal are the estimated immigrants. Values closer to one mean that individuals in that population are mostly a result of self‐recruitment; values closer to zero are closer to all migrants arriving from other populations. In light gray are populations from the Tapajós basin, in dark gray are populations from the Madeira basin. BVI and XAP are from the Cerrado

	TPI	JUR	TAP	GUA	MAM	MAD	BVI	XAP
TPI	**0.85**	0.029	0.013	0.011	0.010	0.016	0.019	0.012
JUR	0.012	**0.911**	0.015	0.011	0.011	0.023	0.013	0.011
TAP	0.013	0.023	**0.871**	0.011	0.011	0.012	0.022	0.011
GUA	0.013	0.020	0.023	**0.791**	0.011	0.183	0.011	0.011
MAM	0.016	0.012	0.013	0.018	**0.677**	0.242	0.011	0.011
MAD	0.017	0.014	0.011	0.011	0.012	**0.814**	0.012	0.011
BVI	0.021	0.035	0.011	0.011	0.01	0.035	**0.845**	0.029
XAP	0.011	0.011	0.012	0.011	0.011	0.011	0.012	**0.917**

BVI, Boa Vista; GUA, Guaporé; JUR, Juruena; MAD, Madeira; MAM, Mamore; TAP, Tapajós; TPI, Teles Pires; XAP, Chapada dos Guimarães.

Overall, our population genetic structure and admixture analyses showed that there is considerable gene flow within the Amazonia resulting in admixed populations, and that the Cerrado populations are separate genetic entities.

## 4 DISCUSSION

### 4.1 Genetic variation is structured within populations of Amazonia

Our results show high genetic variation in Amazonian *M. flexuosa*, seen in the high number of alleles present within populations and no evidence of heterozygote deficiency. This result is consistent with a high degree of polymorphism found in other Amazonian populations of *M. flexuosa* where loci from ISSRs (Rossi et al., 2014) and AFLPs (Gomes et al., 2011) were highly polymorphic. Other outcrossing tropical species such as *Inga* (Fabaceae) species of the Peruvian Amazon (Rollo et al., 2016) and other widespread and abundant palms like *Euterpe precatoria* (Santos et al., 2015) also have high genetic variation.

The finding of high genetic diversity concentrated in Amazonia is congruent with previous hypotheses that parts of this region served as historical refugia for populations of *M. flexuosa* (de Lima et al., 2014). Palaeodistribution models show that *M. flexuosa* populations expanded and contracted during glacial cycling throughout the Neotropics (de Lima et al., 2014; Lima‐Ribeiro, Barberi, & Rubin, 2004), with its fossil pollen record persisting in central Amazonia throughout the Quaternary (Hermanowski, Costa, & Behling, 2012; Hermanowski, Costa, Carvalho, & Behling, 2012).

In the Cerrado, the two populations we sampled have distinct genetic patterns between them (BVI and XAP). The BVI population is the least inbred of these two according to the fixation index (*F*). The area surrounding BVI (Roraima State) is thought to be the “center of origin” for many plant species (Pielou, 1979), including *M. flexuosa* and other palms (Rull, 1998; van der Hammen, 1957). This region is a transition area between dense forests and open areas (de Carvalho & Mustin, 2017), located at the center of the Pleistocene Intertropical Convergence Zone (ITCZ), which is considered an important source area of establishment of different plant species that expanded their ranges during that geological time period (de Lima et al., 2014).

In contrast, the XAP Cerrado population is less diverse and more inbred, which is consistent with previously observed low genetic diversity within populations of *M. flexuosa* in the Cerrado (e.g., de Lima et al., 2014). This is partially explained by our relative smaller sample size as shown by our rarefaction results but may also be due to population decline or incomplete lineage sorting during shifts in forest expanse during glacial cycling. The absence of private alleles in XAP suggests recent population establishment and/or assortative mating. Furthermore, the XAP population is higher in elevation (800 m), with the nearest population at least 300 km away as per our field observations, which suggests high differentiation and lower levels of genetic diversity among populations increased due to high geographic isolation.

### 4.2 Rivers are determinants of *Mauritia flexuosa* population structure

Our population genetic structure and migration analyses suggest that the distribution of rivers is an important factor in the population structure of Amazonian *M. flexuosa*. Populations within basins are connected, while populations among the river basins Tapajós (JUR, TPI, and TAP localities) and Madeira (GUA, MAD, and MAM) have less gene flow. Pairwise immigration estimates show that the MAD population receives immigrants consistent with river flow that moves from the GUA to MAM then MAD localities along the Madeira basin. Overall there is a strong contribution of migrants mostly within basins, supporting the role of exchange within basins as part of the seed dispersal agents in the population diversity and structure of *M. flexuosa*.

We do not discount other means of dispersal including humans (see last section), yet our population genetic structure results are congruent with the fact that rivers are a key element of the landscape in Amazonia. Rivers influence animal distributions such as birds (e.g., Fernandes, Wink, & Aleixo, 2012), frogs (Gascon, Lougheed, & Bogart, 1998), and mammals (e.g., Patton, Da Silva, & Malcolm, 2000) that disperse the seeds of palms and other plants. The impact of rivers on the genetic structure of other plants such as *Myricaria laxiflora* (Tamaricaceae; Liu, Wang, & Huang, 2006) has shown that water flow is a major driver of seed and propagule dispersal, and that migration patterns among populations can form along rivers, similar to what we found here in *M. flexuosa*. Other Amazonian studies have shown that the fruiting of tropical wetland plants occurs in the rainy season when rivers and other bodies of water are overflowed, enabling long‐distance fruit dispersal (De Campos, De Cedro, Tejerina‐Garro, Bayer, & Carneiro, 2013). Rollo et al. (2016) found strong influence of water dispersal in the genetic diversity and structure of *Inga* species in Amazonia. In palms, Oliveira et al. (2014) found that the genetic structure of *Astrocaryum jauari* among different river sites within a river basin with high levels of gene flow within them, likely due to transport of fruits following the direction of the water currents. *Mauritia* swamps are a permanent or temporary shelter for many species of animals that maintain gene flow via seed dispersal (Mendieta‐Aguilar, Pacheco, & Roldán, 2015), and fruits have the ability to undergo long‐distance dispersal and float down rivers, traveling thousands of kilometers, connecting populations at large distances along the water (Moegenburg, 2002).

### 4.3 Insights of influence of human management on genetic diversity and gene flow

Our results are also consistent with the hypothesis suggested that hyperdominant plants in Amazonia, such as *M. flexuosa*, correlate with their proximity to pre‐Columbian archeological sites, and that plant populations of economically important species are maintained preferentially along river margins (Levis et al., 2017). Furthermore, as humans increasingly hunted large vertebrates in forests typically far from the water (Peres, Emilio, Schietti, Desmoulière, & Levi, 2016), animal‐dependent seed dispersal of *M. flexuosa* decreased in those areas, resulting in lower gene flow, all the while maintained closer to rivers. Although these observations remain to be tested explicitly, our patterns of high diversity are also consistent with the hypothesis that large population sizes of this species have been maintained by continuous activities of human cultivation, likely for thousands of years (Levis et al., 2017). As a result, outcrossing would be favored by human tending and a high number of reproductive individuals would be maintained, resulting in a higher effective population size and thus higher genetic variation (Frankham, 1996).

Our data on recent genetic migration among populations also show that the Juruena river (JUR) population, despite being located on the riverbank and having several other populations of *M. flexuosa* nearby, is mostly a result of self‐recruitment (0.91). Unlike other populations in our sampling, this high value could be explained by this area being within an indigenous community, who have used and managed this palm intensively for human consumption and for making handcrafts for hundreds of years (Albernaz‐Silveira, 2016), although this remains to be tested explicitly.

The argument that the distribution of many species, or even the composition of Amazonia, is the result of domestication from pre‐Columbian peoples who altered landscapes for thousands of years has been repeatedly raised by archeologists and anthropologists. Barlow, Gardner, Lees, Parry, and Peres (2012) found a relationship between sites of Amazonian Dark Earth (ancient, anthropogenic fertile soils) and greater plant species diversity and geographic distribution of some species in comparison with sites without anthropogenic effects. Clement (1999), Rull and Montoya (2014), and Thomas, Alcázar Caicedo, McMichael, Corvera, and Loo (2015) have shown that human populations in the Amazon have transformed its physical landscape and transported plant species large distances. The genetic and spatial distribution of *Bertholletia excelsa* (Brazil nut), for example, is strongly linked to areas populated by indigenous groups in Amazonia (Thomas et al., 2015). Rull and Montoya (2014) found the distribution of pollen of *M. flexuosa* linked to millenarian charcoal, suggesting that these came from fires made by local communities for hunting and food preparation.

In the Gran Sabana of Venezuela and likely in the contiguous Roraima savannas of Brazil (e.g., BVI), the gallery forest was more dominant than the savannas 3,100–1,800 Cal yr BP (Leal et al., 2016). The forest was open and disturbed and *Mauritia* pollen was present. From 1,800 Cal yr BP up to the present, savannas ecosystems have been dominant. The synergistic effect between anthropic fires and climate change could have promoted the dominance of savannas. Our results suggest that the BVI population is relatively isolated from the savanna (0.845), and we open the possibility that it has adapted to re‐colonizing habitats disrupted by fire.

The study sites from this work are currently undergoing increasing deforestation and other modifications of forest landscapes. Hydrological connectivity in Amazonia is increasingly disrupted by dynamic and multifaceted drivers (Ritter et al., 2017), including mining, and land‐use changes that have modified at least 20% of Amazonia, with over 150 hydroelectric dams currently in operation and hundreds more planned (Castello & Macedo, 2016). The understanding of the processes related to the maintenance the gene flow throughout different environments, such as that in *M. flexuosa,* could aid conservation and management strategies. Also, the importance of rivers in maintaining population connectivity that are geographically distant is here shown for *M. flexuosa*, which can act as an umbrella for associated species and the environmental that thrive with it. Our results from *M. flexuosa* may be used as a first step toward building a model for other studies of plants whose dispersal is heavily influenced by rivers.

## CONFLICT OF INTEREST

None declared.

## AUTHORS CONTRIBUTIONS

M.T.P., A.B.R., C.J.D.S., and A.C.J. conceived the research. N.L.S., J.C.A., and M.A.A.B. conducted the fieldwork. N.L.S., A.P.V., and M.S.B.B. conducted the lab work. N.L.S. and F.P.Z. did the analyses. C.D.B. and A.C.J. wrote the manuscript with all authors contributing and approving the final manuscript.
