# Supplementary figures and images for: Rivers shape population genetic structure in Mauritia flexuosa (Arecaceae)
Source: Ecol Evol. 2018 Jun 11;8(13):6589–98. doi: 10.1002/ece3.4142 (PMC6053585; doi:10.1002/ece3.4142)

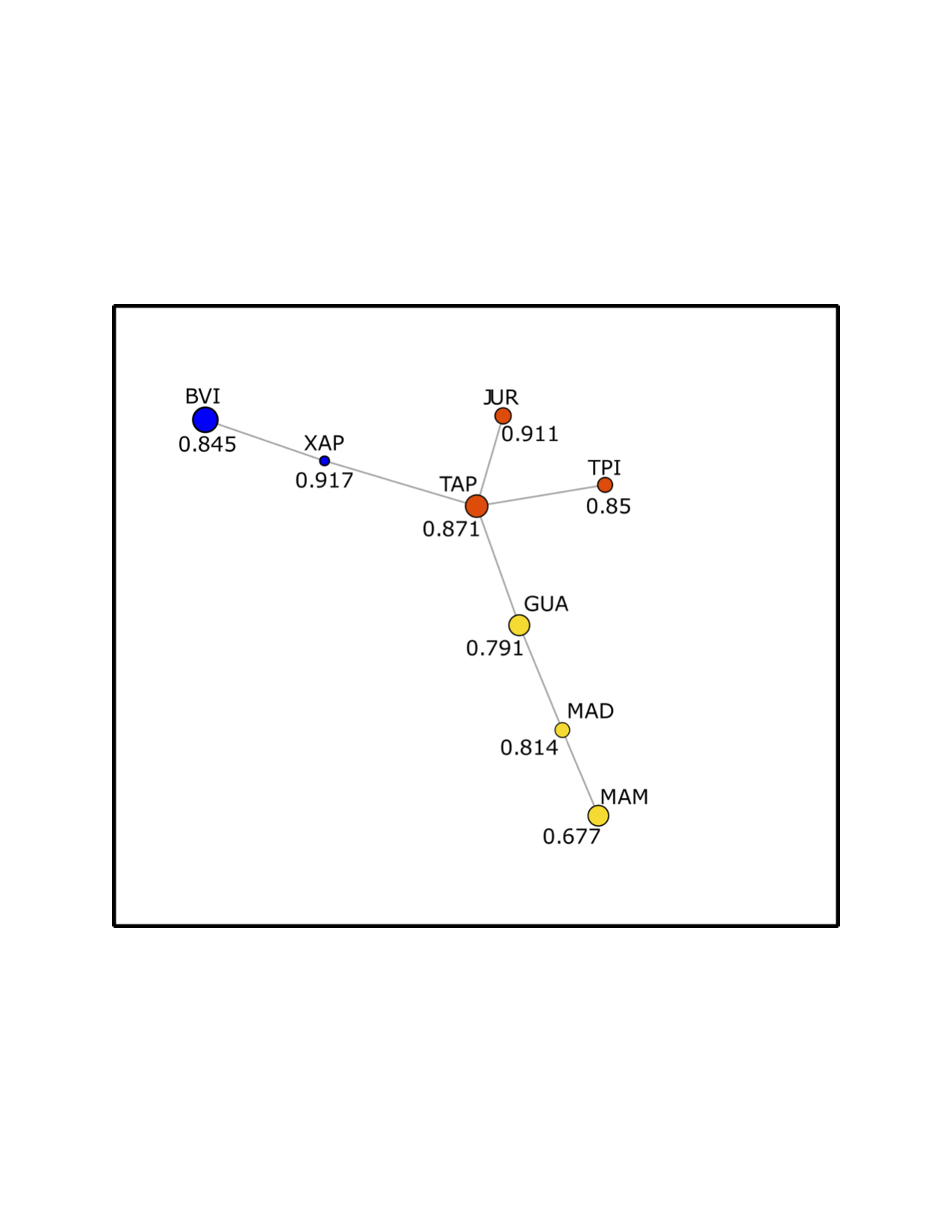

Supplement: Supplementary file 1 [file ECE3-8-6589-s001.jpg]
