# Supplementary material for: Rivers shape population genetic structure in Mauritia flexuosa (Arecaceae)
Source: Ecol Evol. 2018 Jun 11;8(13):6589–98. doi: 10.1002/ece3.4142 (PMC6053585; doi:10.1002/ece3.4142)
Supplement: Supplementary file 2 [file ECE3-8-6589-s002.docx]

**Supplementary Table 1.** Microsatellites generated for *Mauritia flexuosa* L.f.

| Locus | Repeat | Primer sequence (5ꞌ-3ꞌ) | Size range (bp) | | P(Simul *F*st<sample *F*st) | |
| --- | --- | --- | --- | --- | --- | --- |
| **MF9**^1^ | (AG)19 | F: ATACATCGCGCATATCTCACTG | | 113-215 | 0.71 |  |
|  |  | R: ATTCCCACACTCCCTCACTAGA | |  |  |  |
| **MF11**^1^ | (AG)17 | f: AGAGATTGGGGAGGGGAAG | | 103-195 | 0.95 |  |
|  |  | R: TCTCCCTCTCTCTTTCGTTGTC | |  |  |  |
| **MF14**^1^ | (AGA)11 | F: CGGGATAGGAGGTTCAGTGTAG | | 110-276 | 0.96 |  |
|  |  | R: CTCCACCTCTTTGTCTGATTCC | |  |  |  |
| **MF17**^1^ | (AG)7(AG)8 | F: AGGGCTTCTGGAAGTGTCATAG | | 155-191 | 0.99 |  |
|  |  | R: TCCTCTTCTTCTCTCCCTCTTG | |  |  |  |
| **Mf13**^2^***** | (CT)14 | F: TTACAAGCGACCCCTCGTC | | 230–264 | 0.99 |  |
|  |  | R: CGTCGAATAGGGTTTCAGTGG | |  |  |  |
| **Mf14**^2^ | (TC)22 | F: TAGGTCCTGCTTCTGTGCC | | 233–275 | 0.16 |  |
|  |  | R: TGGATCCGGTCCGTTGATAG | |  |  |  |
| **Mf22**^2^ | (CT)15(GT)17 | F: GCATGGTGTAGCTCGTATCTG | | 226–276 | 0.17 |  |
|  |  | R: CGCACCATACTTGGCTTGC | |  |  |  |
| **Mf25**^2^ | (CT)17 | F: CCCCATTTTCCAATTTGATGCG | | 199–225 | 0.34 |  |
|  |  | R: TGGATGTTCAGTTTGGATGCC | |  |  |  |
| **Mf24**^2^ | (TC)20 | F: TCACATTAGTAGTCAAGGGTAGC | | 189-215 | 0.02 |  |
|  |  | R: GGGTGTTAAGCATTCGGGC | |  |  |  |
| **Mf28**^2^ | (GA)9(GG) (GA)11 | F: TCCCACACTCTCTTGCCAC | | 184–200 | 0.34 |  |
|  |  | R: TGAGGGCTGCGTTATGGTC | |  |  |  |
|  | | | |  |  |  |

*Positive selection as measured by P; References: ^1^Menezes et al., 2012; ^2^Federmen et al., 2012.
