# Supplementary material for: Rivers shape population genetic structure in Mauritia flexuosa (Arecaceae)
Source: Ecol Evol. 2018 Jun 11;8(13):6589–98. doi: 10.1002/ece3.4142 (PMC6053585; doi:10.1002/ece3.4142)
Supplement: Supplementary file 4 [file ECE3-8-6589-s004.docx]

| **Supplementary Table 3.** Allele Frequencies per population | | | | | |  |  |  |  |
| --- | --- | --- | --- | --- | --- | --- | --- | --- | --- |
|  |  |  |  |  |  |  |  |  |  |
| **Locus** | **Allele/n** | **JUR** | **TPI** | **TAP** | **GUA** | **MAM** | **MAD** | **BVI** | **XAP** |
| **P3-1** | **N** | 24 | 22 | 22 | 22 | 22 | 21 | 23 | 21 |
| (MF9) | **1** | 0.000 | 0.000 | 0.000 | 0.000 | 0.068 | 0.000 | 0.000 | 0.000 |
|  | **2** | 0.000 | 0.000 | 0.045 | 0.000 | 0.000 | 0.000 | 0.152 | 0.000 |
|  | **3** | 0.167 | 0.182 | 0.136 | 0.045 | 0.045 | 0.000 | 0.000 | 0.000 |
|  | **4** | 0.063 | 0.000 | 0.000 | 0.000 | 0.000 | 0.000 | 0.065 | 0.286 |
|  | **5** | 0.167 | 0.250 | 0.068 | 0.045 | 0.091 | 0.048 | 0.065 | 0.119 |
|  | **6** | 0.250 | 0.227 | 0.159 | 0.159 | 0.386 | 0.524 | 0.283 | 0.048 |
|  | **7** | 0.104 | 0.205 | 0.000 | 0.523 | 0.318 | 0.357 | 0.130 | 0.214 |
|  | **8** | 0.208 | 0.136 | 0.250 | 0.136 | 0.091 | 0.048 | 0.065 | 0.095 |
|  | **9** | 0.021 | 0.000 | 0.091 | 0.091 | 0.000 | 0.000 | 0.152 | 0.071 |
|  | **10** | 0.021 | 0.000 | 0.250 | 0.000 | 0.000 | 0.024 | 0.087 | 0.167 |
| **P4-1** | **N** | 24 | 21 | 22 | 20 | 21 | 20 | 23 | 20 |
| (MF11) | **1** | 0.000 | 0.024 | 0.000 | 0.000 | 0.000 | 0.000 | 0.000 | 0.000 |
|  | **2** | 0.000 | 0.000 | 0.000 | 0.000 | 0.000 | 0.000 | 0.022 | 0.175 |
|  | **3** | 0.000 | 0.000 | 0.000 | 0.000 | 0.000 | 0.000 | 0.022 | 0.000 |
|  | **4** | 0.000 | 0.024 | 0.045 | 0.025 | 0.000 | 0.000 | 0.022 | 0.175 |
|  | **5** | 0.458 | 0.714 | 0.205 | 0.150 | 0.357 | 0.500 | 0.435 | 0.000 |
|  | **6** | 0.042 | 0.095 | 0.000 | 0.100 | 0.143 | 0.175 | 0.087 | 0.000 |
|  | **7** | 0.229 | 0.000 | 0.091 | 0.200 | 0.048 | 0.100 | 0.087 | 0.400 |
|  | **8** | 0.208 | 0.143 | 0.591 | 0.300 | 0.167 | 0.075 | 0.261 | 0.000 |
|  | **9** | 0.000 | 0.000 | 0.023 | 0.175 | 0.071 | 0.075 | 0.000 | 0.025 |
|  | **10** | 0.021 | 0.000 | 0.045 | 0.000 | 0.024 | 0.025 | 0.065 | 0.200 |
|  | **11** | 0.000 | 0.000 | 0.000 | 0.050 | 0.143 | 0.050 | 0.000 | 0.025 |
|  | **12** | 0.042 | 0.000 | 0.000 | 0.000 | 0.000 | 0.000 | 0.000 | 0.000 |
|  | **13** | 0.000 | 0.000 | 0.000 | 0.000 | 0.024 | 0.000 | 0.000 | 0.000 |
|  | **14** | 0.000 | 0.000 | 0.000 | 0.000 | 0.024 | 0.000 | 0.000 | 0.000 |
| **P6-1** | **N** | 23 | 21 | 22 | 21 | 22 | 20 | 22 | 20 |
| (MF14) | **1** | 0.000 | 0.000 | 0.000 | 0.000 | 0.045 | 0.000 | 0.000 | 0.000 |
|  | **2** | 0.065 | 0.238 | 0.136 | 0.000 | 0.000 | 0.000 | 0.000 | 0.000 |
|  | **3** | 0.000 | 0.000 | 0.000 | 0.000 | 0.023 | 0.000 | 0.000 | 0.000 |
|  | **4** | 0.000 | 0.024 | 0.068 | 0.000 | 0.000 | 0.000 | 0.318 | 0.375 |
|  | **5** | 0.435 | 0.000 | 0.000 | 0.000 | 0.000 | 0.000 | 0.045 | 0.000 |
|  | **6** | 0.087 | 0.119 | 0.045 | 0.000 | 0.023 | 0.000 | 0.000 | 0.075 |
|  | **7** | 0.000 | 0.000 | 0.000 | 0.000 | 0.000 | 0.050 | 0.000 | 0.000 |
|  | **8** | 0.000 | 0.000 | 0.068 | 0.000 | 0.000 | 0.000 | 0.000 | 0.000 |
|  | **9** | 0.022 | 0.000 | 0.000 | 0.000 | 0.000 | 0.000 | 0.000 | 0.000 |
|  | **10** | 0.217 | 0.143 | 0.091 | 0.143 | 0.386 | 0.375 | 0.205 | 0.075 |
|  | **11** | 0.065 | 0.000 | 0.000 | 0.000 | 0.000 | 0.000 | 0.000 | 0.000 |
|  | **12** | 0.109 | 0.119 | 0.091 | 0.333 | 0.341 | 0.225 | 0.386 | 0.275 |
|  | **13** | 0.000 | 0.214 | 0.091 | 0.214 | 0.136 | 0.250 | 0.000 | 0.000 |
|  | **14** | 0.000 | 0.143 | 0.341 | 0.310 | 0.045 | 0.050 | 0.000 | 0.000 |
|  | **15** | 0.000 | 0.000 | 0.023 | 0.000 | 0.000 | 0.025 | 0.000 | 0.000 |
|  | **16** | 0.000 | 0.000 | 0.045 | 0.000 | 0.000 | 0.025 | 0.045 | 0.200 |
| **P7-1** | **N** | 23 | 21 | 22 | 21 | 22 | 21 | 22 | 20 |
| (MF17) | **1** | 0.022 | 0.000 | 0.045 | 0.000 | 0.000 | 0.000 | 0.136 | 0.200 |
|  | **2** | 0.065 | 0.000 | 0.000 | 0.143 | 0.000 | 0.048 | 0.182 | 0.175 |
|  | **3** | 0.130 | 0.143 | 0.023 | 0.190 | 0.364 | 0.452 | 0.000 | 0.000 |
|  | **4** | 0.022 | 0.310 | 0.114 | 0.452 | 0.477 | 0.405 | 0.273 | 0.000 |
|  | **5** | 0.022 | 0.000 | 0.182 | 0.048 | 0.000 | 0.000 | 0.000 | 0.000 |
|  | **6** | 0.630 | 0.476 | 0.523 | 0.167 | 0.091 | 0.095 | 0.409 | 0.625 |
|  | **7** | 0.109 | 0.048 | 0.114 | 0.000 | 0.000 | 0.000 | 0.000 | 0.000 |
|  | **8** | 0.000 | 0.024 | 0.000 | 0.000 | 0.000 | 0.000 | 0.000 | 0.000 |
|  | **9** | 0.000 | 0.000 | 0.000 | 0.000 | 0.068 | 0.000 | 0.000 | 0.000 |
| **P11-1** | **N** | 23 | 21 | 22 | 21 | 21 | 21 | 23 | 18 |
| (Mf13) | **1** | 0.087 | 0.048 | 0.045 | 0.119 | 0.071 | 0.048 | 0.217 | 0.833 |
|  | **2** | 0.174 | 0.286 | 0.000 | 0.048 | 0.000 | 0.095 | 0.304 | 0.111 |
|  | **3** | 0.457 | 0.119 | 0.000 | 0.143 | 0.500 | 0.405 | 0.130 | 0.000 |
|  | **4** | 0.130 | 0.500 | 0.432 | 0.405 | 0.286 | 0.071 | 0.174 | 0.000 |
|  | **5** | 0.000 | 0.000 | 0.136 | 0.286 | 0.048 | 0.214 | 0.152 | 0.000 |
|  | **6** | 0.130 | 0.000 | 0.295 | 0.000 | 0.095 | 0.143 | 0.000 | 0.000 |
|  | **7** | 0.000 | 0.000 | 0.000 | 0.000 | 0.000 | 0.024 | 0.022 | 0.000 |
|  | **8** | 0.022 | 0.000 | 0.045 | 0.000 | 0.000 | 0.000 | 0.000 | 0.000 |
|  | **9** | 0.000 | 0.048 | 0.045 | 0.000 | 0.000 | 0.000 | 0.000 | 0.056 |
| **P12-1** | **N** | 23 | 21 | 22 | 20 | 21 | 20 | 23 | 20 |
| (Mf14) | **1** | 0.000 | 0.000 | 0.000 | 0.000 | 0.048 | 0.000 | 0.000 | 0.000 |
|  | **2** | 0.500 | 0.357 | 0.477 | 0.225 | 0.167 | 0.200 | 0.370 | 0.425 |
|  | **3** | 0.000 | 0.000 | 0.000 | 0.000 | 0.048 | 0.000 | 0.000 | 0.000 |
|  | **4** | 0.000 | 0.119 | 0.023 | 0.225 | 0.262 | 0.425 | 0.043 | 0.025 |
|  | **5** | 0.000 | 0.000 | 0.023 | 0.100 | 0.048 | 0.000 | 0.000 | 0.000 |
|  | **6** | 0.239 | 0.238 | 0.045 | 0.200 | 0.262 | 0.200 | 0.152 | 0.150 |
|  | **7** | 0.087 | 0.000 | 0.114 | 0.025 | 0.048 | 0.025 | 0.087 | 0.100 |
|  | **8** | 0.130 | 0.095 | 0.068 | 0.025 | 0.048 | 0.000 | 0.087 | 0.075 |
|  | **9** | 0.000 | 0.000 | 0.000 | 0.000 | 0.024 | 0.000 | 0.000 | 0.000 |
|  | **10** | 0.000 | 0.000 | 0.045 | 0.000 | 0.000 | 0.075 | 0.065 | 0.000 |
|  | **11** | 0.022 | 0.119 | 0.068 | 0.000 | 0.024 | 0.075 | 0.087 | 0.000 |
|  | **12** | 0.022 | 0.071 | 0.136 | 0.200 | 0.024 | 0.000 | 0.109 | 0.225 |
| **P15-1** | **N** | 22 | 22 | 21 | 21 | 22 | 21 | 23 | 20 |
| (Mf22) | **1** | 0.000 | 0.023 | 0.000 | 0.000 | 0.000 | 0.000 | 0.000 | 0.000 |
|  | **2** | 0.000 | 0.000 | 0.000 | 0.000 | 0.068 | 0.000 | 0.000 | 0.000 |
|  | **3** | 0.227 | 0.136 | 0.238 | 0.333 | 0.091 | 0.214 | 0.348 | 0.000 |
|  | **4** | 0.023 | 0.205 | 0.048 | 0.000 | 0.045 | 0.167 | 0.087 | 0.050 |
|  | **5** | 0.318 | 0.341 | 0.214 | 0.190 | 0.205 | 0.119 | 0.326 | 0.575 |
|  | **6** | 0.136 | 0.114 | 0.333 | 0.119 | 0.114 | 0.048 | 0.087 | 0.275 |
|  | **7** | 0.045 | 0.091 | 0.024 | 0.048 | 0.091 | 0.214 | 0.022 | 0.000 |
|  | **8** | 0.136 | 0.000 | 0.071 | 0.024 | 0.159 | 0.095 | 0.065 | 0.075 |
|  | **9** | 0.023 | 0.068 | 0.024 | 0.119 | 0.091 | 0.095 | 0.065 | 0.025 |
|  | **10** | 0.068 | 0.000 | 0.048 | 0.024 | 0.000 | 0.000 | 0.000 | 0.000 |
|  | **11** | 0.000 | 0.023 | 0.000 | 0.143 | 0.091 | 0.048 | 0.000 | 0.000 |
|  | **12** | 0.023 | 0.000 | 0.000 | 0.000 | 0.045 | 0.000 | 0.000 | 0.000 |
| **P16-1** | **N** | 23 | 21 | 22 | 21 | 21 | 20 | 24 | 20 |
| (Mf25) | **1** | 0.000 | 0.000 | 0.000 | 0.000 | 0.071 | 0.000 | 0.000 | 0.000 |
|  | **2** | 0.326 | 0.500 | 0.295 | 0.190 | 0.119 | 0.075 | 0.500 | 0.100 |
|  | **3** | 0.130 | 0.048 | 0.227 | 0.405 | 0.190 | 0.275 | 0.042 | 0.325 |
|  | **4** | 0.065 | 0.214 | 0.273 | 0.190 | 0.381 | 0.300 | 0.208 | 0.225 |
|  | **5** | 0.152 | 0.119 | 0.068 | 0.119 | 0.167 | 0.300 | 0.229 | 0.100 |
|  | **6** | 0.174 | 0.000 | 0.091 | 0.024 | 0.000 | 0.000 | 0.000 | 0.050 |
|  | **7** | 0.087 | 0.119 | 0.045 | 0.071 | 0.071 | 0.050 | 0.000 | 0.200 |
|  | **8** | 0.043 | 0.000 | 0.000 | 0.000 | 0.000 | 0.000 | 0.021 | 0.000 |
|  | **9** | 0.022 | 0.000 | 0.000 | 0.000 | 0.000 | 0.000 | 0.000 | 0.000 |
| **P18-1** | **N** | 22 | 22 | 22 | 21 | 22 | 21 | 24 | 20 |
| (Mf24) | **1** | 0.000 | 0.000 | 0.000 | 0.000 | 0.068 | 0.000 | 0.000 | 0.000 |
|  | **2** | 0.545 | 0.545 | 0.591 | 0.571 | 0.432 | 0.500 | 0.542 | 0.525 |
|  | **3** | 0.136 | 0.068 | 0.114 | 0.024 | 0.068 | 0.071 | 0.125 | 0.175 |
|  | **4** | 0.273 | 0.205 | 0.250 | 0.238 | 0.045 | 0.143 | 0.250 | 0.300 |
|  | **5** | 0.000 | 0.136 | 0.045 | 0.071 | 0.023 | 0.024 | 0.042 | 0.000 |
|  | **6** | 0.000 | 0.000 | 0.000 | 0.095 | 0.295 | 0.214 | 0.021 | 0.000 |
|  | **7** | 0.045 | 0.000 | 0.000 | 0.000 | 0.068 | 0.000 | 0.000 | 0.000 |
|  | **8** | 0.000 | 0.045 | 0.000 | 0.000 | 0.000 | 0.048 | 0.021 | 0.000 |
| **P19-1** | **N** | 23 | 21 | 22 | 21 | 22 | 21 | 24 | 19 |
| (Mf28) | **1** | 0.500 | 0.214 | 0.364 | 0.333 | 0.250 | 0.310 | 0.563 | 0.579 |
|  | **2** | 0.217 | 0.262 | 0.477 | 0.286 | 0.295 | 0.524 | 0.083 | 0.421 |
|  | **3** | 0.152 | 0.286 | 0.023 | 0.190 | 0.205 | 0.119 | 0.042 | 0.000 |
|  | **4** | 0.130 | 0.048 | 0.068 | 0.095 | 0.023 | 0.000 | 0.125 | 0.000 |
|  | **5** | 0.000 | 0.024 | 0.068 | 0.095 | 0.227 | 0.048 | 0.188 | 0.000 |
|  | **6** | 0.000 | 0.167 | 0.000 | 0.000 | 0.000 | 0.000 | 0.000 | 0.000 |

**N** = number of individuals
