# Supplementary material for: Rivers shape population genetic structure in Mauritia flexuosa (Arecaceae)
Source: Ecol Evol. 2018 Jun 11;8(13):6589–98. doi: 10.1002/ece3.4142 (PMC6053585; doi:10.1002/ece3.4142)
Supplement: Supplementary file 5 [file ECE3-8-6589-s005.docx]

**Supplementary Table 4.** Pairwise estimates of *F*st and *R*st

| ***F*_ST_** |  |  |  |  |  |  |  |  |
| --- | --- | --- | --- | --- | --- | --- | --- | --- |
|  | **Teles Pires** | **Juruena** | **Tapajós** | **Guaporé** | **Mamoré** | **Madeira** | **Boa Vista** | **Chapada** |
| **Teles Pires** | **0** |  |  |  |  |  |  |  |
| **Juruena** | 0.05075 | **0** |  |  |  |  |  |  |
| **Tapajós** | 0.06092 | 0.07567 | **0** |  |  |  |  |  |
| **Guaporé** | 0.09558 | 0.08128 | 0.07345 | **0** |  |  |  |  |
| **Mamoré** | 0.10008 | 0.07161 | 0.10836 | 0.03673 | **0** |  |  |  |
| **Madeira** | 0.10838 | 0.07877 | 0.12331 | 0.05029 | 0.07680 | **0** |  |  |
| **Boa Vista** | 0.05012 | 0.06085 | 0.07311 | 0.07526 | 0.08103 | 0.10043 | **0** |  |
| **Chapada** | 0.09617 | 0.14511 | 0.10131 | 0.12236 | 0.14934 | 0.16637 | 0.090703 | **0** |
|  |  |  |  |  |  |  |  |  |
| ***R*_ST_** |  |  |  |  |  |  |  |  |
|  | **Teles Pires** | **Juruena** | **Tapajós** | **Guaporé** | **Mamoré** | **Madeira** | **Boa Vista** | **Chapada** |
| **Teles Pires** | **0** |  |  |  |  |  |  |  |
| **Juruena** | 0.05346 | **0** |  |  |  |  |  |  |
| **Tapajós** | 0.06487 | 0.08187 | **0** |  |  |  |  |  |
| **Guaporé** | 0.10568 | 0.08847 | 0.07927 | **0** |  |  |  |  |
| **Mamoré** | 0.11121 | 0.07713 | 0.12152 | 0.03813 | **0** |  |  |  |
| **Madeira** | 0.12155 | 0.08551 | 0.14065 | 0.05296 | 0.0774 | **0** |  |  |
| **Boa Vista** | 0.05277 | 0.06479 | 0.07888 | 0.08138 | 0.08817 | 0.11164 | **0** |  |
| **Chapada** | 0.10641 | 0.16973 | 0.11237 | 0.17556 | 0.17556 | 0.19958 | 0.10746 | **0** |
|  |  |  |  |  |  |  |  |  |
