# Supplementary material for: Rivers shape population genetic structure in Mauritia flexuosa (Arecaceae)
Source: Ecol Evol. 2018 Jun 11;8(13):6589–98. doi: 10.1002/ece3.4142 (PMC6053585; doi:10.1002/ece3.4142)
Supplement: Supplementary file 6 [file ECE3-8-6589-s006.docx]

| **Supplementary Table 5.** AMOVA analysis. | | |  |  |
| --- | --- | --- | --- | --- |
|  |  |  |  |  |
| **Source** | **df** | **SS** | **Variance components** | **% Variation** |
| Among Groups | 3 | 64.17 | 0.15 | 5.13 |
| Among populations with group | 4 | 35.19 | 0.13 | 4.30 |
| Among individual within populations | 171 | 525.12 | 0.37 | 12.38 |
| Within Individuals | 179 | 417.5 | 23.32 | 78.19 |
| **Total** | **357** | **1041.98** | **298.30** | **100** |

The groups were: Madeira (GUA, MAD y MAM), Tapajos (JUR, TAP y TPI) and BVI and XAP.
